# Lenvatinib Plus Immune Checkpoint Inhibitors Improve Survival in Advanced Hepatocellular Carcinoma: A Retrospective Study

**DOI:** 10.3389/fonc.2021.751159

**Published:** 2021-11-16

**Authors:** Xiaozhun Huang, Lin Xu, Teng Ma, Xin Yin, Zhangkan Huang, Yihong Ran, Yong Ni, Xinyu Bi, Xu Che

**Affiliations:** ^1^ Department of Hepatobiliary Surgery, National Cancer Center/National Clinical Research Center for Cancer/Cancer Hospital & Shenzhen Hospital, Chinese Academy of Medical Sciences and Peking Union Medical College, Shenzhen, China; ^2^ Department of Hepatological Surgery, Second People’s Hospital, First Affiliated Hospital of Shenzhen University, Shenzhen, China; ^3^ Department of Hepatobiliary Surgery, National Cancer Center/National Clinical Research Center for Cancer/Cancer Hospital, Chinese Academy of Medical Sciences and Peking Union Medical College, Beijing, China; ^4^ Department of Gastrointestinal & Pancreatic Surgery, National Cancer Center/National Clinical Research Center for Cancer/Cancer Hospital, Chinese Academy of Medical Sciences and Peking Union Medical College, Beijing, China

**Keywords:** hepatocellular carcinoma, lenvatinib, nivolumab, pembrolizumab, survival

## Abstract

**Background:**

Nivolumab and pembrolizumab disrupt the programmed cell death-1 immune checkpoint and display promising efficacy and safety results in advanced hepatocellular carcinoma (HCC). However, the benefits remain limited. The preliminary results of lenvatinib (LEN) combined with immune checkpoint inhibitors (ICIs) reveal that the combinations were well-tolerated and encouraging. This study aimed to analyze the safety and efficacy of LEN plus ICIs in a real-world cohort of patients with advanced HCC.

**Method:**

Between June 4, 2017, and June 30, 2019, 16 patients received LEN plus nivolumab, and 13 patients were treated with LEN plus pembrolizumab, with the confirmed advanced HCC retrospectively analyzed. The clinical parameters, as well as the outcomes, were assessed.

**Results:**

All the patients had Barcelona Clinical Liver Cancer Stage C. LEN with ICIs was used as systemic second-, third-, and fourth-line treatments in seven (24.1%), 14 (48.3%), and eight (27.6%) patients, respectively. At the time of data cutoff, six patients (37.5%) were still receiving LEN with nivolumab, while another six patients (46.2%) were still receiving LEN with pembrolizumab. An objective response was recorded in seven patients (25.9%), while the best overall responses were from one complete response and six partial responses. The 6- and 12-month over survival (OS) rates were 62.6% and 53.7%, respectively. Furthermore, the 6- and 12-month progression-free survival (PFS) rates were 43.5% and 31.8%, respectively. In the subgroup analyses, the 6- and 12-month OS and PFS rates for patients treated with LEN plus nivolumab were 62.5% and 52.1%, respectively, and 43.8% and 30.0%, respectively. The 6- and 12-month OS and PFS rates for patients treated with LEN plus pembrolizumab were 51.3% and 51.3%, respectively, and 49.2% and 49.2%, respectively. A total of 11 (31%) deaths were reported in this study, four of which were attributed to grade 5 adverse events presented as fatal treatment-related hepatitis.

**Conclusion:**

The combination of LEN and ICIs is a promising new strategy for the treatment of HCC patients. However, high-grade hepatic toxicity was observed and further evaluation of this combination is still required.

## Introduction

Hepatocellular carcinoma (HCC) is the most prevalent primary liver cancer and is ranked as the sixth most common neoplasm, as well as the third leading cause of cancer death (1). However, many patients develop recurrence or disease progression after initial curative surgical or locoregional treatment. At present, there are insufficient therapies that can effectively treat patients with advanced stages of HCC (2, 3). For ten years, the only multikinase inhibitor available for patients with unresectable HCC was sorafenib. Checkmate 459, which is a randomized, multicenter, clinical study, showed that the median overall survival (OS) of the sorafenib group was 14.7 months (4).

In recent years, additional agents, including lenvatinib (LEN) and atezolizumab in combination with bevacizumab, have been introduced to the treatment paradigm as first-line alternatives to sorafenib (5, 6). Similarly, second-line treatment has also evolved, with phase III studies RESORCE, CELESTIAL, and REACH reporting the clinical benefits of regorafenib, cabozantinib, and ramucirumab, respectively, over placebo in patients pretreated with sorafenib (7–9). Immune checkpoint inhibitors have also been examined as novel second-line agents in the treatment of HCC with manageable toxicity in a subset of patients (10, 11). However, phase III studies, in the first-line setting *versus* sorafenib and second-line setting *versus* placebo, have failed to meet their primary endpoints (4, 12).

Although these new inhibitors have improved patient survival, the effectiveness of a single drug remains relatively limited. Furthermore, the benefits remain limited and novel treatment strategies for patients with advanced HCC are urgently required. Numerous studies are examining treatment concepts using combinations of LEN with immune checkpoint inhibitors (ICIs), with preliminary results showing that the combinations were well-tolerated and encouraging (13–15). In 104 patients enrolled in the phase Ib trial of LEN plus pembrolizumab, the confirmed objective response rate (ORR) was 46% and median OS was 22 months (13). Similarly, the phase Ib trial of LEN plus nivolumab revealed manageable adverse events (AEs), and a 76.7% ORR was published in the American Society of Clinical Oncology Seminar in 2020 (16). Based on current data, lenvatinib combined with immunotherapy has shown promising antitumor efficacy and tolerable safety in patients with HCC.

This study aimed to examine the safety and efficacy of LEN plus ICIs in a real-world cohort of patients with advanced HCC treated with sorafenib or more systemic treatment.

## Patients and Methods

### Study Design and Participants

The data of patients with advanced HCC treated with LEN plus ICIs between June 4, 2017, and December 30, 2018, were obtained from the National Cancer Center/National Clinical Research Center for Cancer/Cancer Hospital and Shenzhen Hospital. All data, including patient history, laboratory results, and radiological information were collected retrospectively.

The diagnosis of HCC was confirmed by histologically or cytologically diagnosis, excluding fibrolamellar, sarcomatoid, and mixed hepatocholangiocellular carcinoma. Patients were required to have measurable disease as defined by the Response Evaluation Criteria in Solid Tumors (version 1.1; RECIST v1.1). The following were the other eligibility criteria: a Child-Pugh score ≤ 7 points, an estimated life expectancy of at least ≥ 12 weeks, an Eastern Cooperative Oncology Group performance status ≤ 2, an absolute neutrophil count ≥ 1.2 × 10^9^/L, a platelet count ≥ 50 × 10^9^/L, serum bilirubin ≤ 2 mg/dL, aspartate aminotransferase (AST) ≤ 5 times the upper limit of normal (ULN), alanine aminotransferase (ALT) ≤ 5 times the ULN, serum prothrombin time ≤ 18 seconds, serum creatinine ≤ 1.5 times the ULN, and measured or calculated creatinine clearance ≥ 60 mL/minute. Untreated hepatitis C virus (HCV) and hepatitis B virus (HBV) patients were eligible, but they had to be on anti-HBV or anti-HCV suppression for ≥ 1 week before receiving ICIs. Programmed Cell Death-Ligand 1 (PD-L 1) expression by immunohistochemistry and tumor mutational burden by genetic sequencing were not assessed regularly. Patients were excluded if they had prior treated with LEN or any ICIs.

The treatment of LEN combined with ICIs was administrated after multidisciplinary discussion, and the chosen of nivolumab or pembrolizumab was open label and non-randomized. Patients received 12 mg (body weight ≥ 60 kg) or 8 mg (body weight ≤ 60 kg) LEN orally once daily. The ICIs were administered as recommended by the official dosage and safety information. Nivolumab was administered intravenously at 3 mg/kg body weight or a fixed dose of 240 mg every two weeks. Pembrolizumab was administered intravenously at a fixed dose of 200 mg every three weeks. Dose delays were determined based on toxicity. Treatment schedules were modified at the discretion of the treating physician, if necessary.

The study protocol conformed to the ethical guidelines of the 1975 Declaration of Helsinki, as reflected in *a priori* approved by the institution’s human research committee. The written and informed consent obtained from each patient were included in the study.

### End Points and Clinical Assessments

The primary endpoint was the OS rate at 6- and 12-months. Secondary endpoints included AEs, ORR, progression-free survival (PFS). The AEs were assessed using the National Cancer Institute Common Terminology Criteria for Adverse Events (NCI CTCAE; version 4.03). The radiological response was recorded using computed tomography (CT) or magnetic resonance imaging (MRI) at baseline, 6-12 weeks after treatment initiation, and around every 3 months thereafter. The objective response was defined as the proportion of participants with a confirmed complete response (CR) or partial response (PR) assessed with the RECIST v1.1 guidelines using central imaging review (17).

### Statistical Analysis

This study was designed as a retrospective cohort study. Patients were followed until their death or last contact, or date of censoring if their death did not occur by the cutoff date of July 16, 2019. Data on baseline characteristics, radiological tumor response, and side effects were summarized using descriptive statistics. The radiological response and time to progression of patients who had at least one follow-up imaging assessment were evaluated. TTP was defined as the time between the date of first checkpoint inhibitor administration and the date of the first radiologically confirmed tumor progression. Data from patients who died without radiologically confirmed tumor progression were censored at the date of their last radiological assessment. PFS was defined as the time from the date of the first checkpoint inhibitor administration until radiological disease progression or death, whichever came first. Patients who were still alive and without radiologically confirmed progression at the date of last contact or data cutoff were censored. OS was defined as the period from the start of immunotherapy until the date of death. Patients who were still alive at the date of last contact or data cutoff were censored. Survival curves were determined using the Kaplan-Meier method and compared using the log-rank test. Statistical analyses were performed using IBM SPSS Statistics version 24.0 (SPSS Inc., Chicago, IL). *P* < 0.05 was considered significant.

## Results

### Patient Characteristics and Treatment

A total of 29 patients were assessed for eligibility, with all of them taking at least one immunotherapeutic agent combined with LEN. Nivolumab was administered to 16 patients while pembrolizumab was administered to 13 patients. The baseline patient characteristics are summarized in **Table 1**. All the patients had the Barcelona Clinical Liver Cancer (BCLC) Stage C, with 24 (82.8%) patients infected with HBV, two patients infected with HCV, and two patients were infected with both HCV and HBV. LEN with ICIs was used as systemic second-, third-, and fourth-line treatment in 7 (24.1%), 14 (48.3%), and 8 (27.6%) patients respectively, with all patients receiving at least one systemic treatment, such as sorafenib or regorafenib. Furthermore, most patients received local treatment previously, such as hepatectomy, ablation, locoregional radiotherapy, and transhepatic arterial chemotherapy embolization (TACE). The Child-Pugh scores of A, B, C were reported for 79.3%, 13.8%, and 6.9% of patients, respectively. At baseline, 82.8% of patients had macroscopic portal vein invasion and 79.3% had extrahepatic spread.

**Table 1:** Baseline characteristics.

	LEN + NIVO n = 16	LEN + PEM n = 13	All patients n = 29
Age(y), mean ± SD	49 ± 3.87	57.5 ± 4.22	42.5 ± 3.57
Sex			
Male	4	6	10
Female	12	7	19
Aetiology			
Hepatitis B	14	10	24
Hepatitis C	1	1	2
Hepatitis B+C	1	1	2
Other	0	1	1
Prior treatment			
Hepatectomy	9	6	15
Ablation	3	2	5
Loco‐regional (TACE/radiation)	10	11	21
Previous sorafenib	16	13	29
Previous regorafenib	12	10	22
Previous Anlotinib	3	5	8
LEN+ICIs			
Second‐line	4	3	7
Third‐line	9	5	14
Fourth‐line	3	5	8
Macrovascular invasion	13	11	24
Extrahepatic metastasis	13	10	23
Child-Pugh stage			
A	12	11	23
B	2	2	4
C	2	0	2
ECOG PS			
0	13	10	23
1-2	3	3	6
Alpha‐Fetoprotein			
<400 (IU/ml)	6	7	13
≥400 (IU/ml)	9	6	15

TACE, Transhepatic arterial chemotherapy embolization; LEN, Lenvatinib; ICIs, Immune checkpoint inhibitors; BCLC, Barcelona Clinical Liver Cancer Stage.

The median duration of follow-up was 12.0 months (96%CI: 7.5-17.0 months). At the time of data cutoff, six (37.5%) patients were still on treatment with LEN combined with nivolumab, while six (46.2%) were receiving LEN combined with pembrolizumab. The median duration of treatment for ICIs was 10.5 months (95% CI: 7.53-12.97 months), nivolumab was seven months (95% CI: 3.19-11.38 months), and pembrolizumab was one month (95% CI: 0.67-2.5 months). The most common reasons for treatment discontinuation were progressive disease (PD) in 11 (37.9%) patients and serious AEs in five (17.2%). After PD, seven participants went on to receive an alternative treatment: one received a single LEN, five received regorafenib, and one received the PD-L1 immune checkpoint.

**Figure 1:**
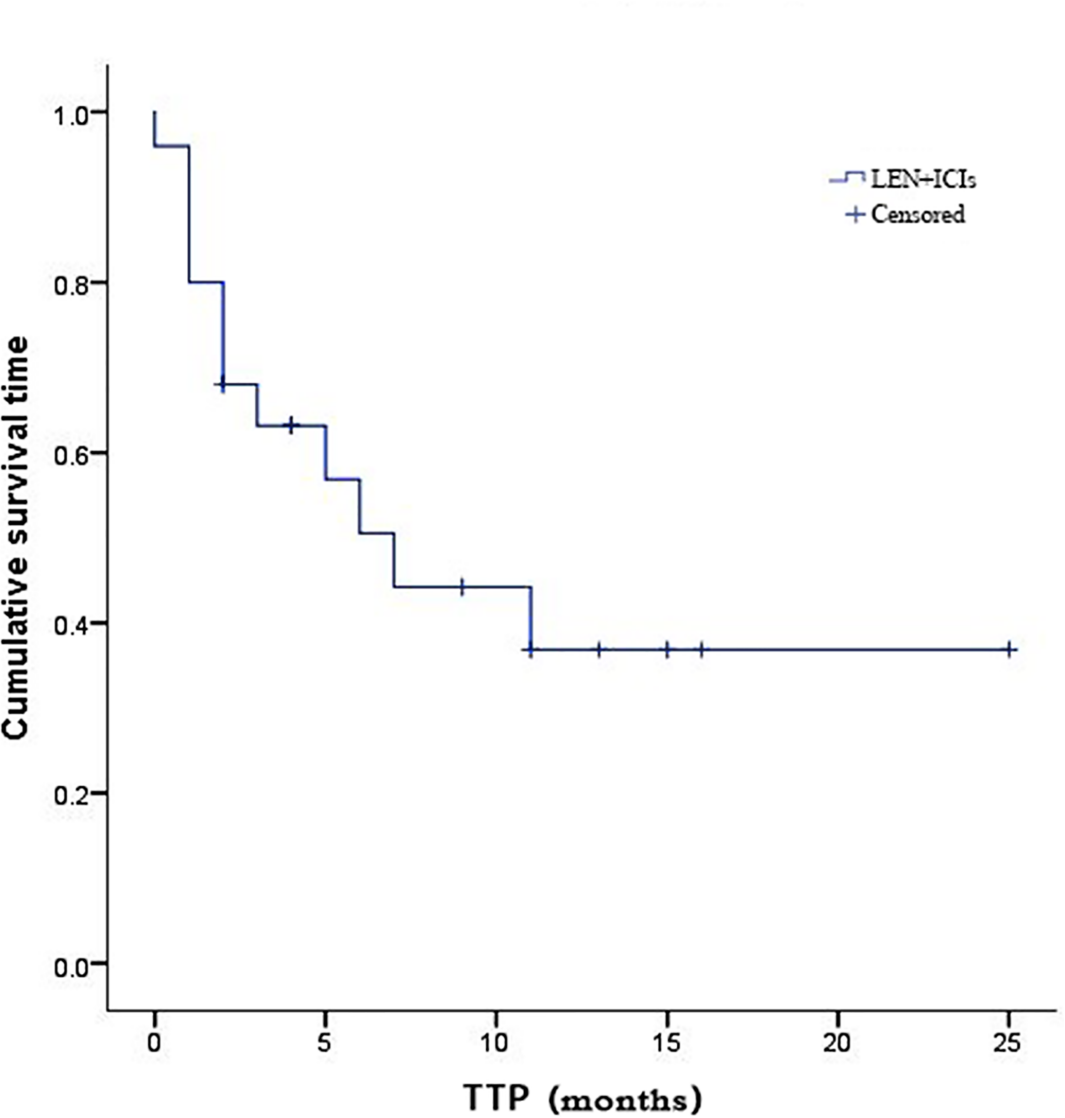
Kaplan-Meier estimates of time to progression for 27 eligible patients with advanced hepatocellular carcinoma who were treated with lenvatinib plus immune checkpoint inhibitors.

**Figure 2:**
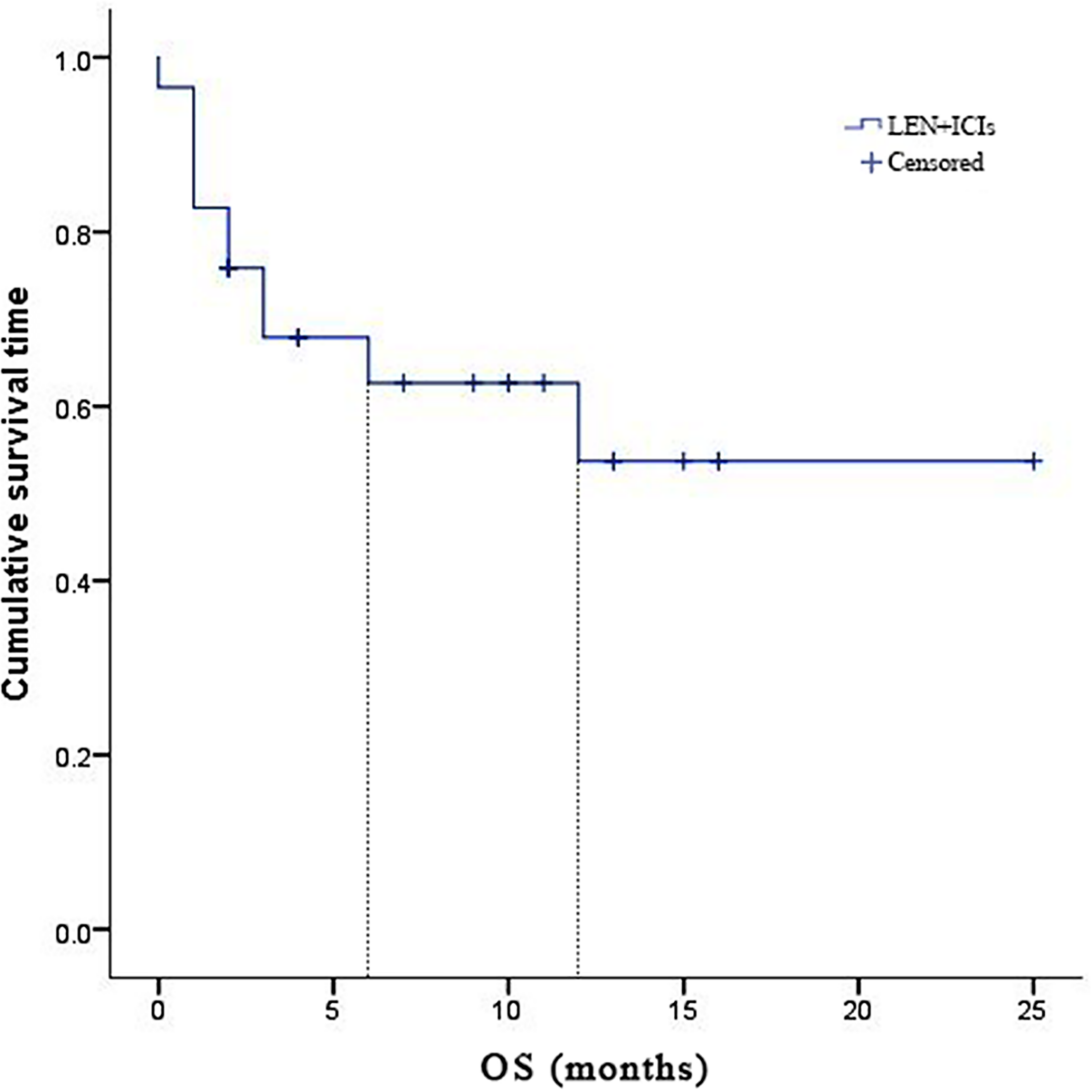
Kaplan-Meier estimates of over survival for 27 eligible patients with advanced hepatocellular carcinoma who were treated with lenvatinib plus immune checkpoint inhibitors.

**Figure 3:**
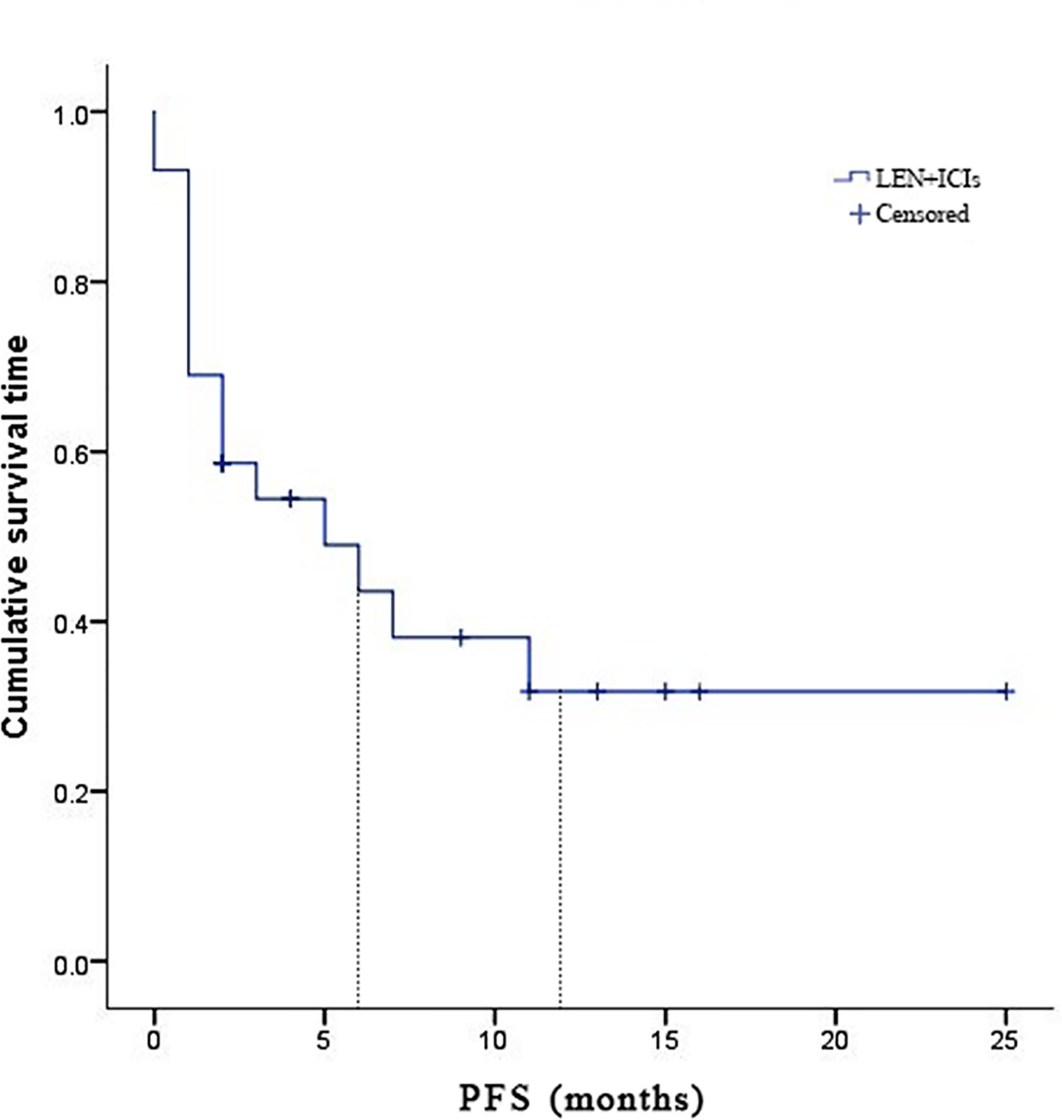
Kaplan-Meier estimates of progression-free survival for 27 eligible patients with advanced hepatocellular carcinoma who were treated with lenvatinib plus immune checkpoint inhibitors.

Due to fatal treatment-related adverse events, two participants in LEN plus nivolumab did not have any assessment data after baseline. An objective response was recorded in seven (25.9%) of the 29 participants who received at least one dose of ICIs. Among the seven responders, the best overall responses were one CR and six PR. Furthermore, 12 (44.5%) participants had stable disease (SD), while eight (29.6%) had PD. The disease control rate (DCR) was reported in 19 (70.4%) of the 27 treated participants (**Table 2**). At the time of data cutoff, six of the seven responses were ongoing, and the median duration of response (DOR) was seven months (95% CI: 1.19-12.81 months). In this study, 11 (40.7%) of the 27 participants died, the median TTP was 7 months (95% CI: 3.44-10.56 months) (**Table 2** and **Figure 1**), the 6- and 12-month OS rates were 62.6%, and 53.7% (**Table 2** and **Figure 2**), respectively, and the 6- and 12-month PFS rates were 43.5% and 31.8% (**Table 2** and **Figure 3**), respectively.

**Table 2:** Radiological response according to RECIST1.1 and survival.

	LEN + NIVO n = 16	LEN + PEM n = 13	All patients n = 29
Best response			
CR	1 (6.3%)	0	1 (3.4%)
PR	5 (31.2%)	1 (7.7%)	6 (20.7%)
SD	4 (25%)	8 (61.5%)	12 (41.4%)
PD	4 (25%)	4 (30.8%)	8 (27.6%)
Not evaluable	2 (12.5%)	0	2 (6.9)
ORR (CR+PR)	6 (37.5%)	1 (7.7%)	7 (24.1%)
DCR (CR+PR+SD)	10 (62.5%)	9 (69.2%)	19 (65.5%)
TTP, median (95% CI)	7 (95% CI 0.39-13.61)	–	7 (95% CI 3.44-10.56)
DOR (range, months)	7 (3-11)	–	7 (3-11)
6-months PFS rate	43.8%	49.2%	43.5%
12-months PFS rate	30.0%	49.2%	31.8%
6-months OS rate	62.5%	51.3%	62.6%
12-months OS rate	52.1%	51.3%	53.7%

CR, complete response; PR, partial response; SD, stable disease; PD, progressive disease; ORR, objective response rate; DCR, disease control rate; TTP, time to progression; DOR, duration of response; PFS, progression-free survival; OS, over survival.

At least one adverse event was reported among the 24 (82.8%) participants: grade 1-2 in 12 (41.4%) patients, grade 3 in six (20.7%) patients, grade 4 in two (6.9%) patients, and grade 5 in four (13.8%) patients (**Table 3**). The following were the most common treatment-related AEs of any grade in the participants: increased ALT concentration in 14 (48.3%), increased AST concentration in 13 (44.8%), and hyperlipemia in 13 (44.8%), nausea in 7 (24.1%), proteinuria in 7 (24.1%), decreased appetite in 7 (24.1%), rash (7 [24.1%]), diarrhea in 6 (20.7%), and asthenia in 6 (20.7%). Treatment-related events of grade 3 or higher severity were reported in 12 (41.4%) participants. The most common grade 3 events were increased ALT concentration, which was observed in 4 patients (13.8%), and elevated AST concentration in three patients (10.3%). Three grade 4 occurrences had allergic reactions, as well as increased ALT and AST concentrations. Among the 29 participants, 4 (13.8%) of them had dose interruptions due to three participants treated with LEN and nivolumab having hepatitis, while the other one treated with LEN and pembrolizumab had severe edema. The three participants in the nivolumab group continued treatment after their hepatitis was cured. Despite the use of systemic corticosteroids for the management of AEs, patients continued to experience clinical benefits. Out of the three patients re-challenged after receiving systemic corticosteroid for AEs, two participants had partial responses, while the third had disease progressive.

**Table 3:** Adverse events.

	LEN + NIVO n = 16		LEN + PEM n = 13		All patients n = 29	
	Any grade	Grade ≥ 3	Any grade	Grade ≥ 3	Any grade	Grade ≥ 3
Rash	3 (18.7%)	–	3 (23.07%)	–	6 (20.7%)	–
Pruritus	2 (12.5%)	–	1 (7.69%)	–	3 (10.34%)	–
Fatigue	3 (18.7%)	–	1 (7.69%)	–	4 (13.79%)	–
Vomiting	3 (18.7%)	–	2 (15.38%)	1 (7.69%)	5 (17.24%)	2 (6.9%)
Diarrhoea	4 (25%)	–	2 (15.38%)	–	6 (20.7%)	–
Paresthesia	–	–	1 (7.69%)	–	1 (3.45%)	–
Arthritis	1 (6.25%)	–	1 (7.69%)	–	2 (6.9%)	–
Thyroiditis	2 (12.5%)	–	1 (7.69%)	–	3 (10.34%)	–
Dyspnea	1 (6.25%)	–	–	–	1 (3.45%)	–
Abdominal pain	2 (12.5%)	1 (6.25%)	–	1 (7.69%)	4 (13.79%)	2 (6.9%)
Nausea	7 (43.75%)	–	3 (23.07%)	–	10 (34.48%)	–
Allergic reaction	–	–	1 (7.69%)	–	1 (3.45%)	–
Gastric ulcer	1 (6.25%)	–	–	–	1 (3.45%)	–
Decreased appetite	5 (31.25%)	1 (6.25%)	2 (15.38%)	–	7 (24.14%)	–
Hyperlipasaemia	8 (50%)	–	5 (38.46%)	–	13 (44.8%)	–
Asthenia	4 (25%)	1 (6.25%)	2 (15.38%)	–	6 (20.7%)	–
Myelosuppression	2 (12.5%)	–	–	–	2 (6.9%)	–
Amylase/Lipase increase	1 (6.25%)	–	–	–	1 (3.45%)	–
AST increase	10 (62.5%)	6 (37.5%)	3 (23.07%)	2 (15.38%)	13 (44.8%)	8 (27.6%)
ALT increase	11 (68.75%)	7 (43.75%)	3 (23.07%)	2 (15.38%)	14 (48.28%)	9 (30.03%)
Proteinuria	4 (25%)	–	3 (23.07%)	1 (7.69%)	7 (24.14%)	1 (3.45%)

AST, aspartate aminotransferase; ALT, alanine aminotransferase.

In this study, 11 (31%) deaths were reported in the study, four of which were attributed to grade 5 adverse events that resulted in fatal immune-related hepatitis. The median time of fatal toxic effects typically occurred in 0.5 ± 1.89 months and the median time from symptom onset to death was five days (range, 1-9 days). Liver protection and prednisone therapy failed to reverse the liver injury, and the dysfunction progressed to liver failure.

**Figure 4:**
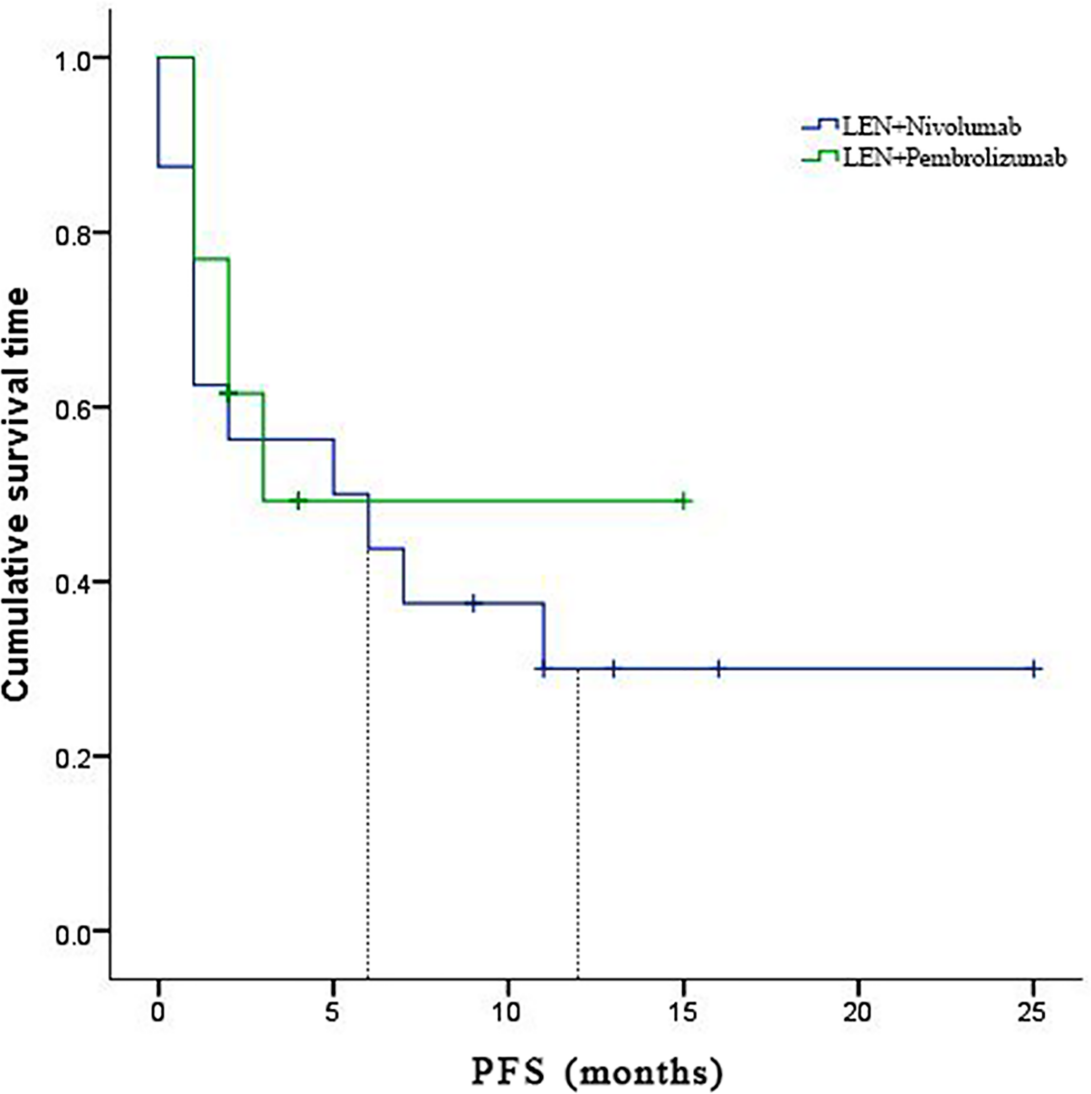
Kaplan-Meier estimates of over survival with advanced hepatocellular carcinoma who were treated with lenvatinib plus Nivolumab and pembrolizumab, respectively.

**Figure 5:**
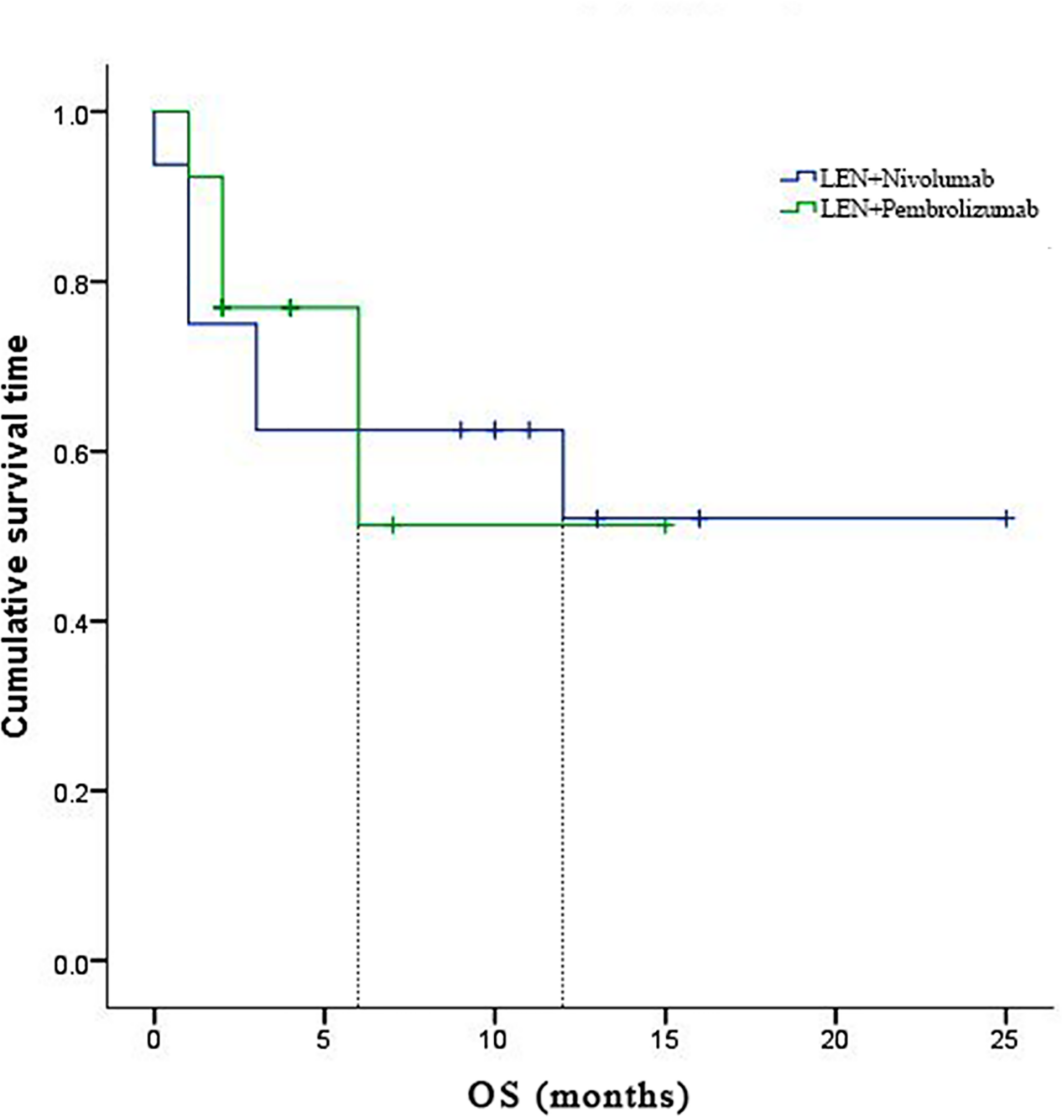
Kaplan-Meier estimates of progression-free survival with advanced hepatocellular carcinoma who were treated with lenvatinib plus Nivolumab and pembrolizumab, respectively.

In the subgroup analyses, ORR was represented in six (42.8%) of nivolumab and one (7.7%) of pembrolizumab. LEN plus nivolumab had the best ORR with one CR (7.14%), five PR (35.7%), and four SD (28.6%). LEN plus pembrolizumab had only one PR (7.7%) and 8 SD (61.5%). DCR was reported in ten (71.4%) of the LEN plus nivolumab group and nine (69.2%) of the LEN plus pembrolizumab group, respectively (**Table 2**). The 6- and 12-month PFS rates for patients treated with LEN plus nivolumab were 43.8% and 30.0%, while for patients treated with LEN plus pembrolizumab they were 49.2% and 49.2%, respectively (**Figure 4**). The 6- and 12-month OS estimates for the LEN plus nivolumab group were 62.5% and 52.1%, respectively, and 51.3% and 51.3%, respectively, for the LEN plus pembrolizumab group (**Figure 5**). In terms of safety, the number of patients who developed any grade (Group nivolumab *vs* pembrolizumab, n = 15 [93.8%] *vs* n = 9 [69.2%]) or high-grade (Group nivolumab *vs* pembrolizumab, n = 7 [43.8%] *vs* n = 5 [38.5%]) adverse events was similar between LEN plus nivolumab or pembrolizumab, with both groups having the same adverse reaction spectrum (**Table 3**).

## Discussion

In second-line trials involving patients who have failed sorafenib, the OS in the placebo group is around 8 months (9, 18, 19). The sequential molecular targeting agent treatment further improved prognosis (20), but late line patients had a worse status and more complex tumor resistance, leading to poor survival during progression after previous systemic therapy. Therapeutic decisions for late-line patients are mainly determined based on the tumor stage and the underlying liver dysfunction.

Several single options evaluated the efficacy and safety of late line HCC compared to the best supportive care or placebo (12, 21, 22). For example, LEN prolonged OS, offering safety and tolerability in first-line treatment (5), as well as providing a good sequential treatment option after progression in the third line of unresectable HCC patients with better hepatic reserve function (21). Furthermore, nivolumab was found to be safe in patients with Child-Pugh class B liver dysfunction (22). In addition, pembrolizumab has demonstrated encouraging antitumor activity and was well tolerated in the Asian subgroup when used as a second-line treatment for advanced HCC (23).

Although a significant number of patients had objective responses and median PFS and OS that were both promising after treatment with LEN or ICIs, the benefits remain limited. Numerous ongoing studies are examining regimens combining LEN with ICIs, with preliminary results revealing that the treatment was well-tolerated and encouraging. There is currently no late-line data on advanced HCC using the combination of LEN and ICIs.

In this study, the combination of LEN with programmed cell death protein 1 (PD-1) targeted immunotherapy demonstrated promising clinical efficacy in a real-world cohort of patients with advanced HCC. A substantial number of objective responses (24.1%) and a DCR of 65.5% were discovered in the 29 treated participants, who were consistently observed across several risk factors associated with the prognosis of advanced HCC. The responses were generally positive; the 6- and 12-month OS rates were 62.6% and 53.7%, respectively; median TTP was seven months (95% CI 3.44-10.56 months); the 6- and 12-month PFS rates were 43.5% and 31.8%, respectively. These findings indicated that the combination of LEN with nivolumab or pembrolizumab could provide an effective treatment option in a late-line systemic therapy setting. In preclinical murine models, the combination of LEN with the anti-PD-1 antibody has been shown to enhance antitumor activity. LEN significantly decreased the population of tumor-associated macrophages, as well as increased the percentage of activated CD8+ T cells secreting interferon-γ+ and granzyme B (24, 25). In addition, LEN significantly reduced the level of tumor programmed death-ligand 1 (PD-L1) and Treg differentiation, improved anti-PD-1 efficacy by blocking FGFR4, and inhibiting TGFß signaling (26, 27). The extent to which combination therapies pose clinical safety and tolerability challenges, and whether these challenges will limit their usefulness as an anticancer therapy, have been the focus of an increasing number of studies.

Recently, the preliminary results of LEN combined with nivolumab or pembrolizumab were reported in first-line treatment evaluating the safety and effectiveness in advanced HCC. The combination of LEN and nivolumab showed a promising ORR of 76.7% and DCR of 96.7% by modified RECIST (16), and the safety was assessed in another trial (28). Meanwhile, the combination of LEN and pembrolizumab showed an encouraging ORR of 36%, and DCR of 88% by RECIST v1.1 (13). In the subgroup of our study, LEN combined with nivolumab had an objective response of 37.5%; DCR was 62.5%; the 6- and 12-month PFS rates were 43.8% and 30.0%, respectively; the 6- and 12-month OS rates were 62.5% and 52.1%, respectively. A combination of LEN and pembrolizumab had a 7.7% ORR and a 62.5% DCR, while the 6- and 12-month overall survival estimates were 51.3% and 51.3%, respectively. Despite the poor prognosis of this population, six patients (20.7%) experienced durable and ongoing confirmed radiographic responses, including one patient who had a complete response at the time of the last follow-up. Future studies assessing the PD-1 score and next-generation tumor sequencing may help in identifying markers of potential responders. In this study, the combination of lenvatinib plus ICIs improved both disease control and survival.

There were no new or unexpected toxicities resulting from the combination of lenvatinib with nivolumab or pembrolizumab (13, 16, 28). The number of discontinuations due to treatment-related AEs was 13.8%, and treatment-related events, such as increased ALT or AST concentration, hyperlipemia, nausea, proteinuria, decreased appetite, rash, diarrhea, and asthenia (events that typically occur following treatment), were observed in more than 10% of participants. Although >80% of subjects experienced AEs, the majority of them were associated with complications of comorbid liver dysfunction and advanced tumor burden, as previously reported in studies on patients with Child-Pugh class B HCC (29). Treatment-related grade ≥3 events were reported to have occurred in 41.4% of patients.

Among the three of the five patients who received systemic corticosteroid for AEs when re-challenged; two had partial responses, while the other had disease progression. The incidence of AEs with immunotherapeutic agents indicated an active immune status, suggesting that there were potential clinical benefits to the patient (30).

In the largest retrospective evaluation of fatal ICIs-associated toxic effects published by the World Health Organization (WHO) pharmacovigilance database (Vigilyze), hepatitis accounted for around 20% of deaths of reported anti-PD-1/PD-L1 related fatalities (31). In the Checkmate-040 study, 22–30% of the patients receiving nivolumab had an increase in ALT/AST levels. A similar rate was also described in the Keynote-224 study of pembrolizumab. This further validated the recently published data of nivolumab in Child-Pugh B patients, where treatment of related hepatic AEs was described in only four out of 49 patients, resulting in treatment discontinuation of two patients in this cohort (32). The most common grade 3/4 immune-mediated AEs in this cohort was liver toxicity, with four deaths attributed to grade 5 AEs presenting fatal treatment-related hepatitis. It was discovered that these events generally occurred very early on after therapy initiation and the duration from symptom onset to death was short; nevertheless, it was unclear how the rates of fatal toxic effects contributed to the combination with lenvatinib. Due to the extremely high prevalence of ICI usage, more aggressive combinations that are in development will cause an increase in life-threatening and fatal complications. Therefore, the potential increased risk of liver toxicity must be taken into account in clinical management.

Despite the retrospective nature and the lack of a control group, the strength of this study is the provision of unique real-world data on multiple lines of a systemic pretreatment patient cohort that is excluded from clinical trials. These findings contribute new, important information on LEN plus ICIs in advanced HCC, particularly the first subgroup report on LEN plus nivolumab.

There are several limitations to this study. Firstly, this study is of a retrospective nature which could influence patient selection bias. Therefore, the results must be interpreted with caution due to the heterogeneous nature of the study population and different treatment regimens. Secondly, the size of the cohort samples was relatively small, reducing the quality of the conclusions reached. Thirdly, due to a lack of detection of PD-L1 expression on tumor cells, future studies will require the evaluation of the PD-1 and PD-L1 expression levels on tumor-infiltrating lymphocytes as potentially valuable biomarkers. In addition, a longer follow-up is required for more meaningful median overall survival results in the cohorts. Finally, the study was not designed to statistically compare the clinical outcomes of lenvatinib plus nivolumab against lenvatinib plus pembrolizumab, and further studies in larger populations are warranted.

## Conclusion

The combination of immunotherapy and targeted therapies has attracted a huge amount of interest in the field, increasing hopes that novel, effective therapeutic options will become soon available, leading to new strategies for the management of HCC patients. However, high-grade hepatic toxicity was observed, which required further evaluation of this combination.

## Data Availability Statement

The original contributions presented in the study are included in the article/supplementary material. Further inquiries can be directed to the corresponding authors.

## Author Contributions

XH, ZH, LX, and XC contributed to conception and design of the study. TM, YR, YN, and XY contributed to the acquisition, analysis, or interpretation of data for the work. XH and XB wrote the first draft of the manuscript. All authors contributed to the article and approved the submitted version.

## Funding

This study was supported by Sanming Project of Medicine in Shenzhen (No.SZSM202011010) and Shenzhen High-level Hospital Construction Fund.

## Conflict of Interest

The authors declare that the research was conducted in the absence of any commercial or financial relationships that could be construed as a potential conflict of interest.

## Publisher’s Note

All claims expressed in this article are solely those of the authors and do not necessarily represent those of their affiliated organizations, or those of the publisher, the editors and the reviewers. Any product that may be evaluated in this article, or claim that may be made by its manufacturer, is not guaranteed or endorsed by the publisher.
